# The scourge of antimicrobial resistance: Containing a global crisis

**DOI:** 10.4102/ajlm.v13i1.2645

**Published:** 2024-11-29

**Authors:** Rajiv T. Erasmus, Chikwelu L. Obi, Sajini Souda

**Affiliations:** 1 Department of Biomedical Sciences, Cape Peninsula University, Cape Town, South Africa; 2 Division of Chemical Pathology, Faculty of Medicine and Health Sciences, Stellenbosch University, Cape Town, South Africa; 3 School of Science and Technology, Sefako Magkatho Health Sciences University, Pretoria, South Africa; 4 Department of Pathology, University of Botswana, Gaborone, Botswana

## Introduction

With the introduction of antibiotics in the 1940s, infectious diseases, which had dominated the field of healthcare for centuries, became treatable and deaths preventable. But today, antibiotic use is being threatened by antimicrobial resistance (AMR) – bacteria, viruses, fungi, and parasites that are difficult to defeat using available antibiotics. Antimicrobial resistance is a normal process that occurs progressively due to genetic changes in pathogens, driven primarily by the misuse and overuse of antimicrobials in humans, animals and plants.^1^ Antimicrobial resistance has become a global threat. In the first comprehensive assessment of the global burden of AMR from 1990 to 2021, it was estimated that 4.71 million (95% uncertainty interval: 4.23–5.19) deaths were associated with bacterial AMR, including 1.14 million (95% uncertainty interval: 1.00–1.28) deaths attributable to bacterial AMR.^2^

Worldwide, antibiotic use increased by 65% between 2000 and 2015, largely associated with overconsumption among developing countries with rising incomes. While the highest antibiotic consumption rates in 2000 were in the United States, France, Spain, New Zealand, and Hong Kong, by 2015, the four top consumers of antibiotics were in low- to middle-income countries, such as Turkey, Tunisia, Algeria, and Romania.^3^ In the Global Antimicrobial Resistance and Use Surveillance System (GLASS) 2022 report that highlighted bacterial pathogen resistance rates, the median stated rates in 76 countries were 42% for third-generation cephalosporin-resistant *Escherichia coli* and 35% for methicillin-resistant *Staphylococcus aureus*.^4^ This is concerning illustratively, when considering that in urinary tract infections caused by *E. coli*, 1 in 5 cases exhibited reduced susceptibility to standard antibiotics such as ampicillin, co-trimoxazole, and fluoroquinolones in 2020.^4^ Africa will be home to 2.5 billion people by 2050.^5^ The United Nations Environment Program^6^ estimated that 10 million people will die of AMR-related causes across the world, and the World Health Organization reported that 4.1 million of these people will reside in Africa.^7^ According to Abubakar and Salman,^8^ the prevalence of antibiotic use among hospitalised patients in Africa varied from 27.6% to 83.5%, with the greater prevalence across West Africa (51.4% – 83.5%) and North Africa (79.1%) when compared to East Africa (27.6% – 73.7%) and South Africa (33.6% – 49.7%).

## Factors contributing to the rise of antimicrobial resistance

Antibiotics are not just used to counter infections in humans. They are also used to maintain the health of animals and crops. The primary cause of AMR in humans is the inappropriate use of antimicrobials. Research shows that 30% of all antibiotics prescribed in United States acute-care hospitals are either unnecessary or inappropriate.^9^ Additionally, antibiotics are prescribed in doctor’s offices and emergency departments for infections that do not need them (e.g. colds and influenza). Antibiotic resistance also spreads easily across the globe today, due to the ease and affordability of travel. The United States Centers for Disease Control Prevention reports that 1 billion people travel across international borders each year, and 350 million travellers arrive in the United States through more than 300 ports of entry.^10^ A resistance threat from anywhere could spread quickly simply by people travelling across borders. The increase in AMR worldwide is also caused by animal and environmental contributors. Antibiotics are regularly added to animal feed and drinking water to prevent sickness. Increases in AMR will make treating infections in animals more difficult and cause animal infections to be more severe,^3^ with ripple effects on zoonotic diseases.

## Economic burden of antimicrobial resistance

The World Bank Group’s final report on drug-resistant infections of 2017 indicated that AMR was more damaging in low-income countries, where the gross domestic product may be eroded by more than 5% of the base case.^11^ In an optimistic low-AMR setting, the modelled losses of global output may exceed $1 trillion per year and, after 2030, may reach $2 trillion annually by 2050.^11^ The report also highlights that this may imply a reduction in other aspects of population well-being, because resources that could have been devoted to reducing poverty or other health-related challenges may need to be re-routed to financing the additional costs of the increased burden of disease. Antimicrobial resistance doesn’t just contribute to healthcare costs – it also impacts levels of productivity. The United States Centers for Disease Control Prevention estimates that annual losses in productivity will cost the United States $35 billion a year.^3^ In low-income countries, AMR not only reduces productivity levels but also shrinks workforces because of associated sickness and premature death.^3^

Challenges, including regular access to clean water and good sanitation, exacerbated by poverty, coupled with the endemicity of HIV/AIDS, enhance the risk of infection and subsequent AMR,^12^ with coronavirus disease 2019 further compromising healthcare infrastructures. The high rates of resistance to commonly prescribed and dispensed antibiotics across sub-Saharan Africa are further worsened by high rates of inappropriate prescribing and dispensing of antimicrobials, weak diagnostic capabilities, variable implementation of regulations concerning the dispensing of antimicrobials without a prescription, and variable access to effective healthcare.^12^ Other compounding factors that add to the challenges of rising AMR rates in sub-Saharan Africa include the availability of substandard or falsified or fake antibiotics. This arises from currently weak regulatory systems, limited local manufacturing, and inadequate quality assurance testing of antimicrobials, as well as concerns with available professionals and co-operation between professional groups.^12^ Some traditional diagnostics, such as growing cultures to identify infectious pathogens, can take too much time. As a result, clinicians may be forced to prescribe antimicrobials before test results are completed, which can lead to overuse and misuse of antibiotics.

## Limited availability of new antimicrobials

The development of new antimicrobial treatments has dropped dramatically since the 1990s and is reaching a crisis stage today. According to the World Health Organization, there were only 27 new antibiotics for priority pathogens in clinical development in 2021, down from 31 in 2017.^13^ In 2019, the Pew Charitable Trusts^14^ documented worldwide efforts to develop new antibiotics. They identified 43 antibiotics in development: four with new applications, 19 that could treat infections caused by some gram-negative bacteria, 10 each that could address urgent threats from gonorrhoea or *Clostridium difficile*; only one in four of these was a novel drug.^14^ New drug development faces daunting hurdles, including lengthy pathways to approval, high costs of development, and low success rates. To address the current AMR crisis, developing a more robust pipeline of new antimicrobials, especially from medicinal plants and animals, must become a priority.

## Laboratories’ role in antimicrobial resistance

Microbiology laboratories provide reliable systems, equipment, and technologies to obtain meaningful data that can be used to detect and monitor infections, AMR, and the efficacy of different antibiotic treatment options. Microbiology and virology laboratories play critical roles in reporting susceptibility studies, which are important in assessing the susceptibility of pathogens to specific antibiotics. Additionally, they support the detection of changes in antibiotic resistance over time. Microbiologists can assist clinicians in providing diagnostic stewardship through identifying specific infections, interpreting test results, and guiding antibiotic treatment. They also assist in identifying susceptibility patterns and conducting research to expand advanced and rapid diagnostic testing methods, as well as contributing to data collection and examining new evidence for the appropriate use of antibiotics. By researching novel antimicrobial compounds, laboratory professionals assist in expanding the antibiotic pipeline

## Conquering antimicrobial resistance: From global priority to your priority

Stopping the spread of AMR demands aggressive actions to prevent or minimise infections worldwide. Measures may include improved antibiotic use, antibiotic prescription, monitoring of AMR genes, uncovering new AMR genes, and the use of antimicrobials in farm animals. Efforts over the past decade have shown that the disciplined, multidisciplinary approach that characterises antimicrobial stewardship can drive measurable improvements. Infection prevention and control, vaccinations, and environmental interventions also contribute to tackling AMR. But success depends on:

- a relentless commitment to action from healthcare leadership
- high levels of engagement by a wide variety of healthcare clinicians and professionals
- data collection and sharing on a local, national, and international basis
- continuous creation and refinement of evidence-based best practices
- collaboration between healthcare providers and public health officials
- extensive education and training within healthcare, across the community
- timely development of novel diagnostic tools and new antibiotic options.

Monitoring of antibiotic use, surveillance, awareness and education, community efforts, developing tools for detection and monitoring, as well as developing dashboards, are strategies that can be used to contain AMR. Rapid diagnostics offer quicker turn-around times and greater accuracy, but as new technologies, they are more expensive and require training and mastery for the effective interpretation of results. Vaccines are also a key preventative measure to limit future infectious diseases and any subsequent inappropriate antimicrobial use with implications for the development of AMR. Vaccines are also less likely to induce resistance.^12^

Each healthcare professional must accept responsibility, underpinned by commitment, to tackle the menace of AMR.

## Conclusion

Tackling the challenges posed by AMR requires a multi-pronged approach, including commitment of healthcare professionals, education, and awareness campaigns on the drawbacks of the indiscriminate use of antibiotics in humans, animals and plants, inappropriate antimicrobial prescribing by healthcare workers, and monitoring of the genes coding for AMR. The concept of ‘one health’ may be threatened by the ripple effects of AMR. Adequate budgetary allocation for the clinical management of AMR, research on development of new antimicrobials, and laboratory diagnostics or investigations of the array of factors responsible for AMR, including sentinel surveillance, are critical imperatives in addressing the conundrum of AMR.
